# An Account of Several Cases of General Diseases Cured by the Extraction of Decayed and Diseased Teeth: In a Letter to Dr. Edward Miller, of New-York, from Benjamin Rush, M. D. &c.

**Published:** 1803-11-01

**Authors:** 


### *An* ACCOUNT *of several* CASES *of general* DISEASES *cured by the* EXTRACTION *of decayed and diseased* TEETH : *In a Letter to Dr.* EDWARD MILLER, *of New-York, from* BENJAMIN RUSH, *M. D. &c.*

SOME time in the month of October, 1801, I attended Miss A. C. with a rheumatism in her hip-joint, which yielded, for a while, to the usual remedies for that disease. In the month of November it returned with great violence, accompanied with a severe tooth-ach. Suspecting the rheumatic affection was excited by the pain in her tooth, which was decayed, I directed it to be extracted. The rheumatism immediately left her hip, and she recovered in a few days. She has continued ever since to be free from it.

Soon after this I was consulted by Mrs. J. R. who had been affected for several weeks with dispepsia and tooth-ach. Her tooth, though no mark of decay appeared in it, was drawn by my advice. The next day she was relieved from her distressing stomach complaints, and has continued ever since to enjoy good health. From the soundness of the *external* part of the tooth and the adjoining gum, there was no reason to suspect a discharge of matter from it had produced the disease in her stomach.

Some time in the year 1801 I was consulted by the father of a young gentleman in Baltimore, who had been affected with epilepsy. I inquired into the state of his teeth, and was informed that several of them in his upper jaw were much decayed. I directed them to be extracted, and advised him afterwards to lose a few ounces of blood at any time when he felt the premonitory symptoms of a recurrence of his fits. He followed my advice ; in consequence of which I had the pleasure of hearing from his brother that he was perfectly cured.

I have been made happy by discovering that I have only added to the observations of other physicians, in pointing out a connection between the extraction of decayed and diseased teeth, and the cure of general diseases. Several cases of the efficacy of that remedy, in relieving head-ach and vertigo, are mentioned by Dr. Darwin. Dr. Faber relates that Mr. Pettit, a celebrated French surgeon, had often cured intermitting fevers which had resisted the bark for months, and even years, by this prescription ; and he quotes from his works two cases, the one of consumption, the other of vertigo, both of long continuance, which were were suddenly cured by the extraction of two decayed teeth in the former, and of two supernumerary teeth in the latter case.

In the second number of a late work, entitled, " Bibliotheque Germanique Medico-Chirurgicale," published in Paris, by Dr. Bluver and Dr. Delaroche, there is an account, by Dr. Siebold, of a young woman who had been affected for several months with great inflammation, pain and ulcers in her right upper and lower jaws, at the usual timeof the appearance of the catamenia, which, at that period, were always deficient ih quantity. Upon inspecting the seats of those morbid affections, the Doctor discovered several of the molares in both jaws to be decayed. He directed them to be drawn, in consequence of which the woman was relieved of her monthly disease in her mouth, and afterwards had a regular discharge of her catamenia.

These facts, though but little attended to, should not surprise us when we recollect how often the most distressing general diseases are brought on by very inconsiderable inlets of morbid excitement into the system. A small tumour, concealed in a fleshy part of the leg, has been known to bring on epilepsy. A trifling wound with a splinter or a nail, even after it has healed, has often induced a fatal tetanus. Worms in the bowels have produced internal dropsy of the brain, and a stone in the kidney has excited the most violent commotions in every part of the system. Many hundred facts of a similar nature are to be met with in the records of medicine.

When we consider how often the teeth, when decayed, are exposed to irritation from hot and cold drinks and aliments, from pressure by mastication, and from the cold air, and how intimate the connection of the mouth is with the whole system, I am disposed to believe they are often the unsuspected causes of general, and particularly of nervous diseases. When we add to the list of those diseases the morbid effects of the acrid and putrid matters which are sometimes discharged from carious teeth, or from ulcers in the gums created by them, also the influence which both have in preventing perfect mastication, and the connection of that animal function with good health, I cannot help thinking but our success in the treatment of all chronic diseases would be very much promoted by directing our inquiries into the state of the teeth in sick people, and by advising their extraction in every case in which they are decayed It is not necessary that they should be attended with pain, in order to produce disease; for splinters, ters, tumours, and other irritants before mentioned, often bring on disease and death when they give no pain, and are unsuspected as causes of them. This translation of sensation and motion to parts remote from the place where impressions are made, appears in many instances, and seems to depend upon an original law of the animal economy.

